# Lessons in leadership: celebrating Dr. Wilma Bergfeld’s legacy

**DOI:** 10.1097/JW9.0000000000000247

**Published:** 2026-02-11

**Authors:** Thomas N. Helm, Dirk Elston

**Affiliations:** a Department of Dermatology, Pennsylvania State Health, Hershey, Pennsylvania; b Department of Dermatology, Medical University of South Carolina, Charleston, South Carolina

**Keywords:** dermatology, history, impact, leadership, societies

The Cleveland Clinic’s centennial celebration of excellence highlighted the extraordinary career of Dr. Wilma Bergfeld, whose impact on dermatology and dermatopathology has been profound.^1^ Former trainees paid tribute to her trailblazing career, reflecting the breadth of her influence over generations. To date, Dr. Bergfeld has trained 78 dermatopathology fellows along with countless residents and medical students. Her fellows and their fellows have, in turn, trained hundreds of dermatopathology fellows, firmly establishing her worldwide legacy. She continues to see patients, provide expert dermatopathology services, and has served for decades as Chair of the Cosmetic Ingredient Review, ensuring the safety of cosmetic ingredients in consumer formulations. In addition, she devoted many years to the Food and Drug Administration Dermatology Advisory Committee, where she reviewed new dermatologic applications to advance clinical care.

A prolific scholar, Dr. Bergfeld, has authored more than 900 publications. Yet beyond her academic contributions, her true “super talents” have been her ability to mentor, build enduring relationships, and lead at the highest levels of professional dermatology. She has served as President of the Cleveland Dermatological Society, the American Academy of Dermatology, the American Society of Dermatopathology, and the American Dermatological Association—all while maintaining an active clinical and dermatopathology practice at the Cleveland Clinic. She also held leadership roles as President of the Ohio Dermatological Association, the Cleveland Academy of Medicine (where she was the first female President), and the Cleveland Clinic Foundation Staff, in addition to serving on the Clinic’s Board of Governors and Board of Trustees.^2^ In recognition of her extraordinary contributions, she was honored as a Master Clinician by both the American Academy of Dermatology and the Cleveland Clinic Foundation. Perhaps her greatest gift is a talent for mentorship and establishing meaningful personal connections. Her past fellows have remarked that no matter how busy she is, when you need to interact she stops what she is doing, makes eye contact and is totally connected with you and the conversation at hand.

Her personal life is equally inspiring. Sixty-three years ago, Wilma married her medical school classmate, Dr. John Bergfeld. Together they raised 2 children and now cherish time with their grandchildren. John often reflects that no matter how busy Wilma’s professional life became, she always managed to prepare family dinners, join him on sailing trips across the Great Lakes, and provide unwavering support for their family.

Dr. Bergfeld’s dedication to training the next generation of physicians remains a central passion. She expects her trainees to bring flexibility, positivity, and a willingness to learn—qualities rewarded with countless lessons in persistence, hard work, intellectual curiosity, and relationship-building. Her mentees are connected through the vibrant “Demodex Society,” a network that now spans multiple institutions across the globe. The “Demodex 2.0” generation includes leaders at programs such as the Medical University of South Carolina, Penn State Hershey Medical Center, the University of Alabama Heersink School of Medicine, the University of Vermont, and the University of California, Irvine, among many others (Fig. 1). Her global influence has been profound with “Demodex Society” grandchildren and great grandchildren teaching and practicing dermatopathology on every continent. In May, a meeting of the Chinese Demodex Society will take place in her honor in Hangzhou, China, including more than 50 third generation Demodex Society members and streamed virtually to their hundreds of students. Similar tributes are planned around the globe. Wilma has been a pioneer and key mentor for generations of dermatologists and dermatopathologists.

**Fig. 1.**
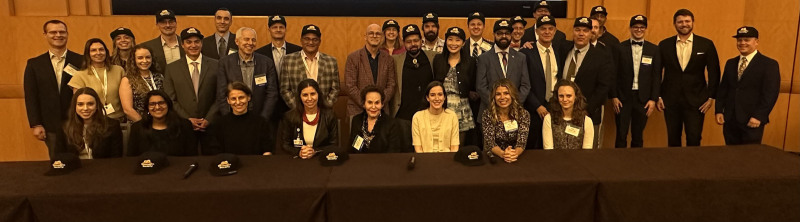
Members of the Demodex Society attending the September 27th “Dermatopathology pearls and reflections from colleagues and members of the Demodex Society” session as part of the “A Century of Dermatologic Excellence at Cleveland Clinic: looking at the Past and Future to Celebrate the Career of Dr. Wilma Bergfeld” at the Intercontinental Hotel and Conference Center, Cleveland, OH.

Dr. Bergfeld has always prioritized her guiding values:

1. Personal principles and integrity.
2. Her marriage.
3. Her children and grandchildren.
4. Her career, colleagues, and trainees.

In an era when many physicians struggle with burnout, s Bergfeld remains energized. Her intellectual curiosity, compassion for patients, and love for dermatology continue to fuel her work and inspire those around her. Her legacy is not only measured in publications and positions held but also in the generations of physicians she has trained and the example she has set as a clinician, leader, and role model.

One of her greatest legacies is the creation of the Women’s Dermatologic Society, an organization that epitomizes the values of dedication, leadership and mentorship that helps to establish networking and collegial support.^3^ Throughout her career, she has exemplified the enduring qualities of fostering relationships and leading by example.

Few have contributed as much to our field as Wilma.

Wilma—You rock!

## Conflicts of interest

None.

## Funding

None.
